# Sampling Scheme Conception for Pretreated Polyolefin Waste Based on a Review of the Available Standard Procedures

**DOI:** 10.3390/polym14173450

**Published:** 2022-08-24

**Authors:** Mohamad Hassan Akhras, Joerg Fischer

**Affiliations:** 1 Competence Center CHASE GmbH, Altenberger Straße 69, 4040 Linz, Austria; 2 Institute of Polymeric Materials and Testing, Johannes Kepler University, Altenberger Straße 69, 4040 Linz, Austria

**Keywords:** waste management, plastics recycling, waste sampling, polyolefin, polyethylene, polypropylene, plastic packaging, high-quality recyclates

## Abstract

Given the rapid development of plastics recycling in recent years, the need for guidelines for sampling and material characterization is steadily emerging. However, there still exists a considerable scarcity of methods that enable proper material data acquisition. This paper consists of two parts. The first part provides a critical review of the available sampling techniques that can be utilized in the field of plastics recycling. Several sampling studies were covered in the review alongside the prominent standardization institutions. It was found that neither the literature nor the standards provide a comprehensive practice that considers the distinctive characteristics of plastic waste and applies it to different situations along the value chain. In the second part, a proposal of a sampling plan for pretreated rigid plastic waste is conceptualized based on selected information from the reviewed methods. Two variants of the proposed plan were evaluated based on the flake size distribution and the apparent density of four different pretreated polyolefin (PO) waste materials. The results of the study showed that combining stratified random sampling with composite sampling yields a good sampling technique for rigid PO waste. Moreover, the analysis of a composite sample adequately conveys the true material properties of a sublot or lot.

## 1. Introduction

The total production of plastics had grown to 368 million metric tons (Mt) in 2019 [1,2]. This number is expected to rise to 460 Mt by 2030 [3]. While, over 50% of the global production of plastics takes place in Asia, Europe accounts for only 16%, coming after China and the countries of the North American Free Trade Agreement (NAFTA) [1,2]. By 2015, the carbon footprint of plastics had increased by a factor of two with respect to the estimations of 1995 [4]. Hence, ample pressure from the public and policy makers has been imposed for solutions towards a more sustainable value chain in the plastics industry. Since the environmental impact of plastics is often lower than that of other alternatives, a total ban of plastic products is definitely disadvantageous [4,5]. It was estimated that the production of plastics and other means of recovery, such as incineration, annually generate around 400 Mt of CO_2_. However, the recycling of plastics can reduce the extraction of fossil fuels and inhibit the greenhouse gas emissions [5,6,7,8]. Therefore, several governmental bodies and policy makers established action plans to emphasize the importance of plastics circularity through reusing and recycling [6,9]. In 2018, the European commission set an ambitious action plan and a strategy to enhance plastics recycling and encourage further developments in this field. The plan aims to recycle over 50% of plastic waste in Europe and to make all packaging plastics either reusable or recyclable by 2030 [6,7]. Furthermore, the plastic circulation strategy, that was introduced by the Japanese government in 2019, is another example of the increasing awareness of plastics circularity. The strategy dictates a complete transition to reusable and/or recyclable plastic packaging by 2025 followed by a target to reuse or recycle 60% of all plastic containers and packaging products by 2030. Moreover, it also aims to fully recycle or reuse all plastic waste by 2035 [9].

Nevertheless, it is essential to understand the life cycle of plastic products of certain applications from material selection to generated waste to increase the recycling rates, close the loop, and produce high-quality recyclates from the plastic wastes [10,11]. In addition, elaborate information about the material properties throughout the value chain of plastics has to be collected through proper characterization procedures, which in turn require reliable and representative sampling techniques [12]. The objective of this research is to contribute to the development of quality control (QC) systems for plastics recycling by providing an overview on the available sampling methods and conceptualize a scheme for the sampling of pretreated rigid polyolefin (PO) waste at plant level. 

### 1.1. Life Cycle of Polyolefin Products

PO materials have become the most widely used polymers in the plastic industry. POs, mainly polyethylene (PE) and polypropylene (PP), dominate the European market covering around 50% of the total plastics demand [1]. The low costs and the diverse property profile of the products made them a perfect fit for packaging applications [13]. Packaging represents the largest share with 39.6% of the total production of plastics in Europe [1]. Consequently, packaging products account for 61% of postconsumer plastic waste. In 2018, 17.8 Mt of postconsumer plastic waste (PCPW) from packaging applications were collected. However, only 42% of the collected waste was recycled [1,14].

**Figure 1:**
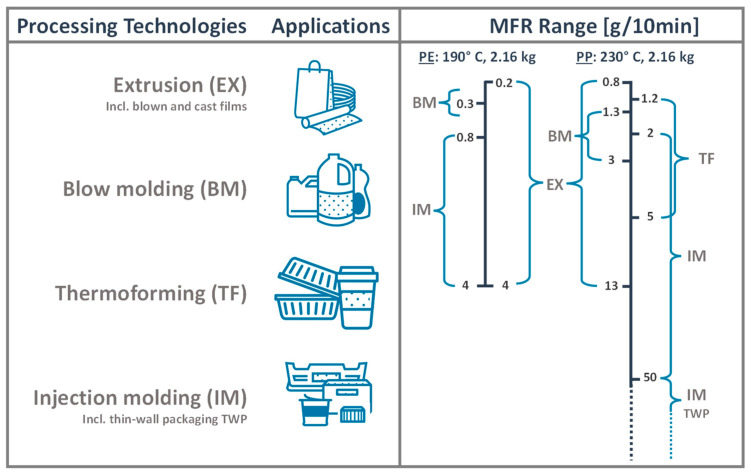
Illustration of the correlation between the MFR values of certain PO grades from packaging applications and the suitable processing technologies. Compiled based on several product data and information sheets from Borealis AG [18,19,20,21] (own illustration).

Plastic packaging can be divided into flexible and rigid packaging. Rigid packaging (RP) includes a wide spectrum of products, such as thin-walled containers, bottles, bottle caps, transport boxes, etc. These products are normally produced by various processing technologies (e.g., injection molding, extrusion, blow molding) and, based on the desired property profile of the end product, proper material selection is required [15]. Melt flow rate (MFR) of the polymer is usually used as an indicator to determine the compatible processing technology for a certain product [11,16,17]. Gall et al. [16] carried out a survey of relevant material selection guides and data sheets from several polymer suppliers to investigate the diversity of materials that are used for cap and closure applications in the beverage industry. In another study, Eriksen et al. [11] presented a link between typical packaging products and suitable processing methods based on the MFR value of various PO grades. Both studies agree that MFR values of different PE and PP types, which are typically used for packaging applications, vary greatly depending on the processing technology as well as the product requirements. According to Erikson et al. and Gall et al. [11,16], the spectrum of MFR values of some packaging products may range from as low as 0.3 up to 30 g/10 min in the case of high-density polyethylene (PE-HD) and it expands to well above 50 g/10 min for PP. Figure 1 illustrates suitable technologies to produce some packaging products and the compatible MFR values of the used PO materials.

The first type of rigid plastic waste is usually generated during the production of RP products. This fraction never reaches the consumer, thus it is referred to as pre-consumer plastic waste, or more widely known as postindustrial plastic waste (PIPW) [22,23]. PIPW typically includes the residues of the production process, such as injection molding runners and sprues [24]. Such waste streams are often composed of mono polymers and not contaminated by other materials, thus they can be recycled easily and are usually used to produce high quality recyclates [25]. On the other hand, PCPW is generated after the intended use and disposal of a product by the end consumer (household or commercial) [22]. Hence, the composition of such waste streams is more complex, since they are contaminated and consist of various polymers that originate from multiple applications and have distinct property profiles (e.g., MFR, color, density) [26].

It is believed that the contamination level of PCPW is highly dependent on the employed collection system and varies from one country to another [25]. In general, there are several collection strategies for PCPW including single-source separation, whereby certain PCPW materials are separated directly by the consumer at disposal or commingled collection, in which all different waste materials are disposed of and collected together [27]. In Austria for instance, two collection schemes for plastic waste are implemented. The first scheme is by collecting all packaging PCPW in the yellow bag/bin after disposal. However, in some regions, only plastic bottles are collected for recycling, whereas the other types of light-weight packaging are collected together with the residual solid waste to be used for energy recovery [28]. Once the plastic waste is collected, it goes to the waste management facilities, where it is sorted and treated to be prepared for the next recovery processes [25,26]. While the above mentioned individual process steps are describing a formal collection system, as typically found in EU countries, in low- and middle-income countries (e.g., in Africa, Asia, Latin America) an informal collection system (ICS) is usually employed [29,30]. Presumably, depending on the specific ICS, the contamination level can vary significantly. However, according to a study carried out by Gall et al. [31], the implemented ICS in Nairobi, Kenya, leads to recyclates with a relatively low contamination level, although it relies on waste pickers. 

There are several recovery paths for the plastic waste, including mechanical recycling, chemical recycling, dissolution, or energy recovery via incineration or re-oiling processes [14]. Usually, the appropriate recovery method is selected depending on the contamination and degradation levels of the plastic waste [32]. However, the current review is mainly focusing on the sampling and characterization of pretreated rigid PO flakes for mechanical recycling.

### 1.2. Mechanical Recycling of Plastic Waste

**Figure 2:**
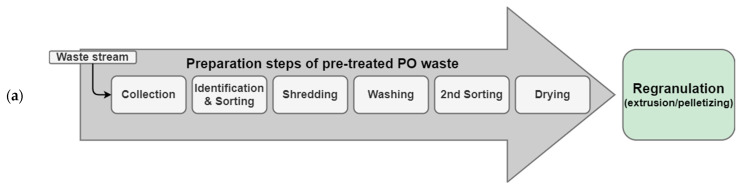

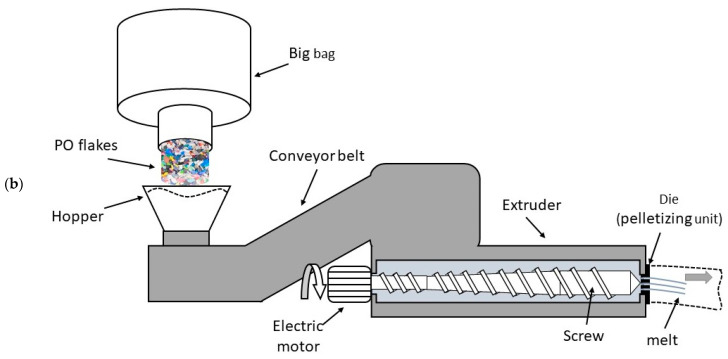
(**a**) Simplified sequence of the preparation steps of the pretreated polyolefin (PO) flakes based on ISO 15270:2008 [38], (**b**) schematic representation of the regranulation process on a machine equipped with a single screw extruder. This representation is inspired by the process set-up at LIT Factory, Linz, Austria (own illustration).

The EU directives of waste management have established new regulations and policies to eliminate landfill and replace it with more sustainable means of disposal and recovery of plastic packaging [33,34]. Consequently, a lot of effort has been made to make plastic production circular and transform it from the old “take-make-dispose” model into the “reduce-reuse-recycle” model. Extending the service life of polymeric materials through reuse and recycling is essential for a circular economy (CE) of plastic packaging [10,14,35]. Amongst the different methods of plastics recovery, mechanical recycling of PCPW is still considered the most desirable approach and the most sustainable practice after reusing [23,36]. Mechanical recycling, or secondary recycling, usually refers to processes that transform the plastic waste into secondary raw materials by physical means (e.g., shredding, extrusion) [32,34]. The regranulation of plastic waste into pellets via extrusion is the most common recycling technology due to its relatively inexpensive operational costs, applicability to different polymers, high efficiency, and the possibility of enhancing the quality of the polymer melt by the addition of degassing and filtration units to the extruder [34,36,37]. In general, the operational sequence of mechanical recycling always starts with the collection of PCPW and ends with the regranulation process. However, certain pretreatment steps, such as sorting, shredding, washing and drying are usually implemented before extrusion to improve the quality of the input materials, thereby the quality of the resulting recyclates [23,38]. Hence, mechanical recycling can, practically, be divided into preparatory steps, whose product is pretreated flakes, and the regranulation process, which converts the flakes into recyclates. However, the order and the repetition of the pretreatment steps may differ depending on the source and the contamination level of the plastic waste [25]. Figure 2 depicts an operational sequence of a mechanical recycling process for polyolefins. However, the process steps are shown in a simplified manner and thus the extrusion process does not involve filtration and degassing.

### 1.3. The Inherent Heterogeneity of Plastic Waste

Despite its high efficiency and relatively low costs, mechanical recycling of PO waste still has several issues to overcome. The major challenge is the low performance of the regranulates in comparison to their virgin counterparts, which limits their applicability to numerous applications and deems them to be used for inferior products [32,39]. This is mainly due to two factors. The first is the possible material degradation, which is induced by the high temperature and shearing during the extrusion process, while the second factor is driven by the heterogeneity of the plastic waste [23,32]. PCPW is substantially heterogeneous and often contaminated by other polymers and chemicals [25], since it is generated from different sources and different products [11]. The high heterogeneity of PCPW is a consequence of several factors that can be summarized as: 1. Using immiscible polymers in the production of plastic products [40]; 2. Various product types that can be produced by different processes, thus higher feedstock variability in regard of the property profile and morphology of the materials [41]; 3. Variety of features in the product designs, such as colors, which increases the complexity of the composition of a waste stream as well as difficulty in sorting certain colors (i.e., black) due to the limitation of the sorting technologies [42,43]; 4. The use of multiple inseparable polymers in some packaging applications (e.g., multilayer films or coffee caps), which always lead to a certain degree of polymer cross-contamination [44,45].

The possibility of producing high-quality recyclates that can substitute virgin materials is highly dependent on the quality of the incoming waste and its ability to meet the requirements of particular applications as well as the operational conditions of the recycling process [46,47,48,49]. In their assessment of the management system of plastic packaging in Austria, the authors of [10] investigated the polymer composition of the waste flow and found that the plastic waste is mainly dominated by PE-LD, PET, and PP. Similarly, the authors of [45] investigated the source of household rigid plastic waste in Denmark to study the composition of such waste streams and to evaluate their degree of heterogeneity. They found that the waste consisted of a variety of packaging products from various applications (e.g., shampoo bottles, beverage bottles, food trays), which are made of different polymers with different designs and colors. Both studies suggested that such compositions are not easily converted into high-quality recyclates. The reason is that these polymers cannot be sufficiently sorted by the available technologies (e.g., density sorting, NIR) because of the similar densities of some polymers (e.g., POs), black pigmentation, and the size and shape of the flakes [23,42]. Consequently, the heterogeneity of the plastic waste will still be present even after the pretreatment processes. Another study was carried out by Luijsterburg and Goossens [50] to examine the impact of the collection system on the composition and selected properties of postconsumer PO recyclates. They found that the collection system did not have a significant impact on the quality of the recyclates. However, the properties of the recyclates were considerably influenced by the sorting and the reprocessing step.

**Figure 3:**
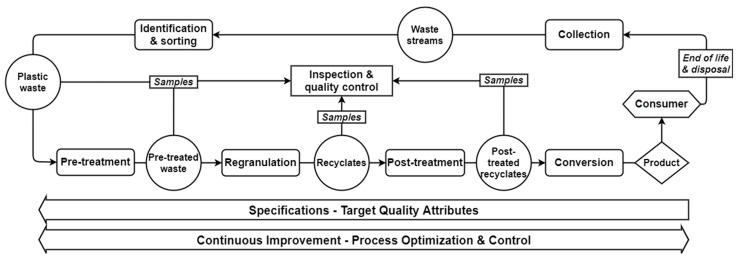
Recycling process of plastic waste together with an indication of reasonable sampling at different stages to enable adequate process control for high-quality recyclates (own illustration).

Therefore, taking into account the specifications of the final product, the collection of detailed information about the composition and the basic properties of the input materials throughout the recycling process is essential to determine the processing parameters and control the quality of the recycled output (Figure 3) [11,48]. A typical recycling process in a recycling plant starts with the pretreatment (e.g., shredding, sorting, washing) of sorted plastic waste. The pretreated plastic waste is transferred into regranulation. Afterwards, the resulting recyclates might undergo some post-treatment steps (e.g., decontamination, compounding) before being converted into new products [38]. Compounding, with virgin materials and/or additives, is usually used to improve the quality of the recycled plastics to fulfil product requirements [17,51]. Several organizations and initiatives introduced guidelines that include sets of requirements and test protocols to evaluate the reliability of the waste supplier as well as the recyclability of the input materials in the recycling industry [52,53]. However, the sampling procedure is often overlooked despite its importance for the generation of reproducible test results that can be used to improve the process and the quality of the end product [12,54]. The overall aim of this paper is to address the importance of sampling for the generation and acquisition of representative material data at the different stages of the recycling process of plastic waste, particularly before the regranulation step.

## 2. Review of the Available Sampling Methods for Plastic Waste

Proper characterization and testing of input materials are crucial for the generation of accurate datasets. These datasets are used to improve the recycling process and technologies, thus, to produce recyclates that meet the quality attributes of specific applications. In general, there is still a lack of information in terms of material characteristics, which creates the need for more studies to be performed in this field [55,56,57]. When it comes to sampling procedures, the gap is even bigger since most of the research is based on the analysis of household waste at the source to provide an overview of the composition of different waste streams in different regions [58,59,60,61]. Such studies are often built from a holistic perspective to deliver statistical analyses for the decision and policy makers without considering the technological and engineering aspects of the plastics recycling process nor the final quality of the recycled materials [62,63].

According to Dahlén and Lagerkvist [58], there is no international or European standard for the sampling and characterization of household waste, which has led to various sorting and sampling approaches and hindered the reproducibility and comparability of results between different studies [59,61]. In their review paper [58], provided an overview of the most common methods in composition studies of household waste in general and analyzed the applicability of the theory of sampling (TOS) for the collection of samples from household waste. They concluded that, for a proper sampling procedure, it is necessary to divide the investigated flow into strata, define the number of samples and sample size, define the sampling location, and to select the component characteristics to be investigated. Another study carried out by Glass and Dominy [64] examined the influence of acceptance sampling before the sorting step on the quality of recycled polyethylene terephthalate (PET). In their study, they focused on the recovery level of polyvinyl chloride (PVC), whose presence deteriorates the quality of recycled PET. They inferred that knowing the PVC level in a feedstock through acceptance sampling combined with the recovery rate of the sorting process helps to predict if the desired quality can be achieved.

Reliable sampling provides information about a whole lot through the inspection of a representative sample with a manageable size. However, only proper unbiased sampling will yield such representative samples [65]. In other words, the sole inspection of a sample is not enough to ensure its representativeness, thus, the whole sampling procedure has to be evaluated [66,67]. There is still no consensus on how to perform proper sampling in the plastics recycling industry to ensure a constant quality of the recycled plastics. For the purpose of this paper, sampling methods from the most prominent norming organizations, including the International Organization for Standardization (ISO); Deutsches Institut für Normung (DIN); American Society for Testing and Materials (ASTM), were selected based on a set of criteria to be reviewed. To be selected, the standards had to be available in English or in German and to explicitly address sampling. Additionally, they had to be directed at plastic waste, waste management, heterogeneous waste, or at bulk materials in general. Table 1 summarizes the sampling methods, which are of relevance to the plastics recycling industry. 

ISO has not yet composed any standardized method for the sampling of plastic waste. However, it established ISO 11648 as a two-part technical practice for the statistical sampling of bulk materials, for which no standards have yet been introduced [68,69]. The first part of the standard [68] outlines the necessary technical terms and statistical methods that can be used for the sampling of bulk materials in general, while the second part provides a guideline of the basic sampling techniques from particulate materials. The aim of this part is to create a consistent approach to attain representative primary increments, from which test samples can be prepared without introducing systematic errors. According to the standard, the operational sequence of the quality inspection of bulk materials usually consists of sampling, sample preparation, and analysis and testing of the sample. To have an acceptable level of precision, four points should be taken into consideration along this sequence. First, the primary increments of the sample should be collected in an unbiased fashion; second, avoid the introduction of systematic errors during sample preparation; third, the relevant quality characteristics should be defined; fourth, performing the appropriate analytical techniques on calibrated equipment according to standardized methods.

**Figure 4:**
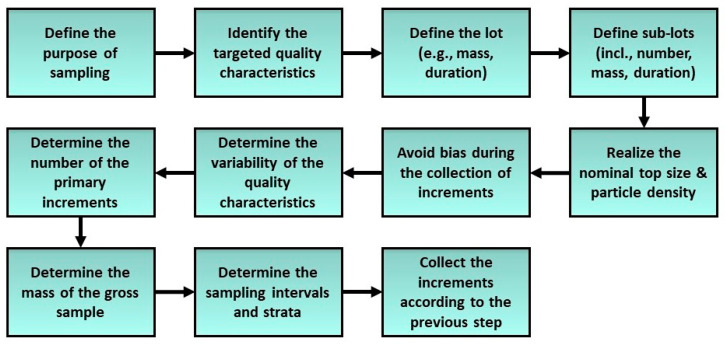
Operational sequence of eleven steps for an unbiased sampling scheme according to ISO 11648–part 2 [69] (own illustration).

To collect unbiased increment samples, all particles of a bulk material must have equal chances to be picked out of the lot. Hence, a reliable sampling scheme reduces the effective bias to zero and thus ensures that the sampling procedure is based on correct selection probabilities [66,69]. Theoretically, bulk materials are always three-dimensional. However, in industrial operations materials are usually described by a one-dimensional model, as the other two dimensions are presumed of less importance and neglected. Thereby, stratified systematic sampling can be carried out for the collection of the primary increments. However, if a systematic error can possibly be introduced, it is strongly recommended to use stratified random sampling within defined intervals instead [69]. Consequently, ISO suggests a sequence of eleven steps to be followed in the development of a sampling practice for regular QC activities (see Figure 4). In certain experimental cases, such as determination of variability, the sample increments can be investigated individually. Nonetheless, in regular sampling, the increments of a lot or a sublot are combined to constitute a gross or composite sample, from which portions are then taken for the sample preparation of the respective test methods [69]. 

**Figure 5:**
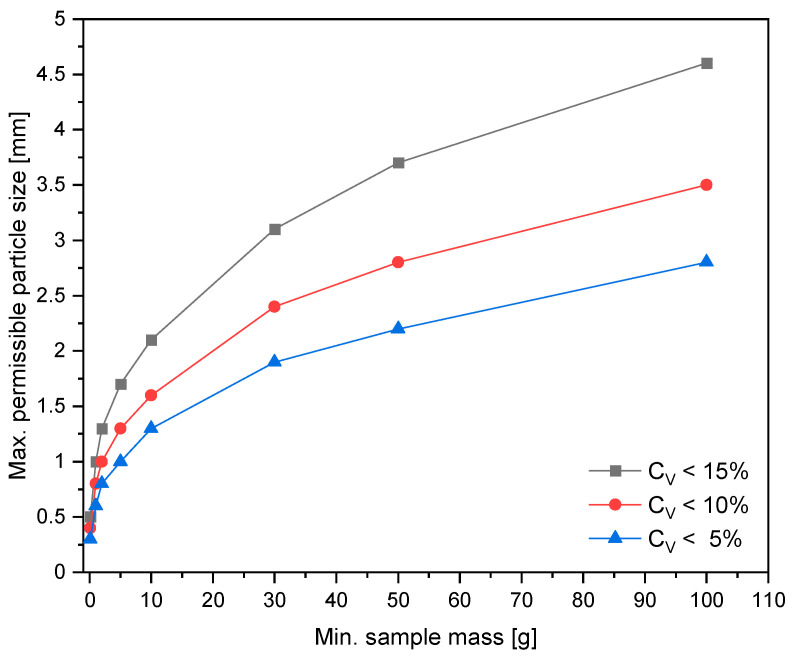
The relationship between the largest particle size and the sample mass with various coefficients of variation (C_V_), compiled by the authors based on the table of minimum masses of a laboratory sample in DIN 16011 [71] (own illustration).

In contrast, the technical committee (TC) for plastics (CEN/TC 249) at the “Deutsches Institut für Normung” (DIN) launched a series of ten publications on plastics recycling. Two of these publications are designed specifically for the sampling and sample preparation of plastic waste and recyclates [70,71]. However, these two standards are primarily derived from ISO 11648 in combination with other sampling standards of other material classes (e.g., metal ore and coal). Their advantage is that they take into consideration the characteristics and conditions of plastic waste and recyclates and provide a simplified sampling approach to the plastics recycling industry. The purpose of DIN 16010 is to set a guideline that enables plastic recyclers to calculate the risk of inaccuracy due to a sampling regime. It also aims to define the basic sampling procedures that must be followed to characterize the sampled material. Hence, it describes a system of sampling methods for testing plastic waste and recyclates and presumably covers the different stages of the recycling process. According to [70] these procedures are expected to lead to representative samples. However, some variations may occur due to certain factors, such as the mixtures of different polymers, origin of the waste, previous application, remaining content from use, moisture, or residual from inert content in the material. Nevertheless, the collected samples must be sufficiently representative of the lot or the batch to generate useful information for the user. Hence, the practice suggests using statistical tools (e.g., t-student distribution) to evaluate the representativeness of the sample and determine the effectiveness of a sampling routine. Moreover, for the establishment of a sampling plan, it recommends following the same operational sequence in accordance with ISO 11648–part 2 (Figure 5).

On the other hand, DIN 16011 [71] defines procedures to be followed in sample preparation for the subsequent testing of various material properties (e.g., chemical, mechanical, thermal). The standard emphasizes that the sample should remain representative after the sample preparation steps. Thus, it must be well mixed and all processes that might lead to segregation should be avoided before the extraction of the laboratory portion. This is particularly necessary when increments or subsamples are collected from different sources and then combined to form a gross or a composite sample. Additionally, the contamination behavior of the sample should be observed carefully to ensure a sufficient level of homogeneity. According to DIN 16011, the determination of the minimum size of a laboratory sample is dependent on the material properties to be measured. In addition, the minimum mass of a laboratory sample is affected by the particle size as it increases with the larger grain size of the single unit (e.g., granulate, flake). Hence, the minimum sample mass is usually determined based on the largest particle size of the material to be analyzed. For this purpose, the standard provides a table of values of various particle sizes with different precision levels for the determination of the mass of a laboratory sample. The relationship between these values is depicted in Figure 5. Furthermore, in the case of test methods that require the production of molded parts (i.e., mechanical tests), the sample preparation is also affected by the physical form of the materials. Therefore, size reduction might be required in the case of flakes and agglomerates. DIN 16011 provides a short guideline on the basics of sample preparation including different techniques and recommendations for size reduction, mixing, and splitting.

Nonetheless, these two technical guidelines only offer broad principles and concepts that should be taken into account when creating a sampling plan for plastic waste and recyclates. They do not, however, offer a concrete sampling procedure that could be implemented in various steps throughout the value chain of plastics recycling. Moreover, they primarily concentrate on the statistical analysis of samples, thus, they are insufficient for routine sample collection and QC activities. However, they can be employed for the quality inspection of incoming materials or waste streams (e.g., acceptance sampling). 

The technical committees (TCs) of the American Society for Testing and Materials (ASTM) have published numerous standards for the sampling of specific materials, although the TC on plastics (D20) has not yet introduced any practice for the sampling of plastic waste nor did the TC on waste management (D34). However, the subcommittees D34.01.01/02 of the D34, whose responsibilities are planning for sampling and sampling techniques, established a complementary suite of 25 standards, which provide guidance and knowledge for the development of a sampling strategy for a variety of waste materials in different states and situations [72,73]. Based on the criteria proposed by the authors, seven of these standards were selected to be reviewed in this paper (Table 1). It is known that each material has its own peculiar characteristics and problems due to certain factors, such as its state; location; degree of homogeneity; size; stratification; or segregation [74]. These factors must be considered in the development of sampling strategy for a certain material type. ASTM D4687 [65] defines a waste sampling plan as “*A scheme or design to locate sampling points so that suitable representative samples descriptive of the waste body can be obtained*”. This practice provides general information on multiple aspects of waste sampling that are present in the most common situations. These aspects include safety, sampling plans, quality assurance, general sampling considerations, preservation and containerization, cleaning, logistics (e.g., packaging, shipping), and chain-of-custody procedure. The advantage of this standard is that it addresses the essential points and requirements to be considered in the formulation of a sampling plan. Nonetheless, it does not provide any comprehensive framework or sampling procedures for any specific applications or waste types.

Generally, ASTM emphasizes the importance of representative samples for a successful sampling plan in all listed standards. Hence, it provides a detailed discussion on the topic of representative sampling for waste management activities in the standard guide ASTM D6044 [75]. This guide defines the representativeness in sampling and describes the attributes that a sample should possess to adequately infer the characteristics of a population. It also outlines the process by which representative samples can be obtained and identifies error sources that should be avoided. The major emphasis in this standard is given to bias, as it has a direct impact on the inference from samples to their populations. The standard differentiates between three sources of bias that must be considered in the sampling design including sampling bias, measurement bias, and statistical bias. However, the practice only provides a theoretical overview of the substantial points and factors that should be considered for obtaining representative samples.

For heterogeneous waste, ASTM D5956 [76] offers a practical guide for sampling strategies, which apply to several applications and material types. The standard merely presents a nonmathematical discussion of the issues related to the sampling of such waste materials and takes into consideration certain population attributes (e.g., degree of heterogeneity). Yet, it is consistent with inferential statistics and the sampling theory of particulate materials; thereby, it can possibly serve as a basis for statistical treatment of sampling problems. The standard thoroughly discusses the different definitions of heterogeneity and stratification from different perspectives with illustrative examples to have a basic understanding of these terms. Moreover, it describes the relationship between samples to populations and how it is reflected in the design of the sampling plan. According to ASTM D5956, the degree of heterogeneity is estimated by the evaluation of the sample attributes, particularly, the representativeness of a population characteristic and the intersample variance. Therefore, the collection of multiple samples by an appropriate sampling scheme is necessary to be able to estimate the degree of heterogeneity of a population and to have a better representation of its properties. This information, in combination with the specifications and the test requirements, can be utilized to optimize the sampling procedure.

**Figure 6:**
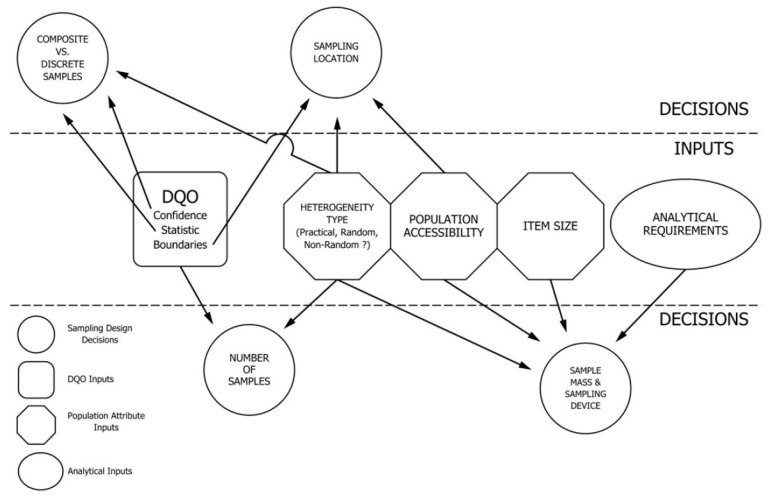
Sampling decisions and sampling design based on data quality objectives (DQO) and population attribute inputs [76].

Figure 6 depicts the essential aspects that need to be considered in a sampling design as well as the critical sampling decisions to which they are related. The use of discrete or composite samples is discussed in multiple standards. A representative sample can be single, composite, or a group of samples [76]. The topic of composite sampling and subsampling for waste management activities is thoroughly discussed in the standard guide [77]. The guide is profoundly focused on solid waste materials, where a reduced amount of laboratory sample is desired. Composite- and subsampling are vital in the sequence of activities that must be performed in accordance with the quality objectives to yield representative data. The use of composite samples can improve the accuracy to estimate the average properties of a population due to a reduced intersample variance. Other advantages gained from composite sampling are its cost-effectiveness and the ability to identify and locate hotspots in populations more efficiently [76,77]. In contrast, the limitations of this method include loss of information of the individual samples as well as the potential dilution of contaminated samples with uncontaminated ones. In general, the standard aims to help those involved in the development of a sampling plan decide which sample type is compatible with the sampled material. Moreover, it also provides an overview of simple mixing procedures (e.g., hand mixing, size reduction, sieving) to homogenize and prepare the composite samples, although, it does not discuss the factors that concern the collection of the individual samples nor the interpretation of the acquired data, as they are covered in other respective guides.

The D34 committee offers several sampling guides that consider the spatial dimensions of a waste population. One of which is the standard guide ASTM D6009 for the sampling of waste piles [78]. This practice presents sampling methods for collecting representative samples from waste piles, such as municipal waste, for the purpose of waste characterization, treatment, or disposal. Furthermore, it addresses the factors associated with the waste body and their impact on the strategic and design consideration of a sampling plan. The standard emphasizes that the development of a sampling strategy for waste piles is highly influenced by the variability of the pile features, especially those that stem from the history of the waste, the physical characteristics of the pile, and the waste properties. For instance, the number of required samples as well as their accessibility are influenced by the variability in the pile size, shape and stability. In terms of waste characteristics, the knowledge of particle size or contaminant distribution reduces the necessary sampling requirements to define the properties of interest. Therefore, this guide takes into consideration these factors to provide an overview of potential sampling strategies and equipment that cover distinct situations of pile waste sampling. However, it only addresses in-place methods including directed sampling, simple random sampling, stratified random sampling, systematic grid sampling, and systematic sampling over time.

In certain situations, sampling from a moving conveyor is more favorable than in-place methods for the evaluation of waste piles as it ensures equal chances of being picked for all material units [78]. Therefore, D34 published a standard practice, ASTM D7204, for waste stream sampling on open and closed conveyors [79]. The practice provides a concise description of procedures for sampling from conveying systems regardless of their movement direction. Moreover, it is directed at particulate materials and slurries that can be scooped or shoveled. However, it is not applicable for large size particles. Although the standard does not make any reference to plastic waste or recycling, the procedures for sampling on a conveyor belt are generally applicable for various applications. Hence, they can be useful for developing a sampling plan for plastic waste recycling.

Finally, ASTM D6311 [80] presents a systematic approach for the selection and optimization of sampling designs for waste management activities. The standard aims to establish a practical guideline for the development steps towards an optimum sampling scheme. This guideline covers the stages from the initial design selection to the final establishment. It also outlines the factors by which the selection process is influenced, such as the performance characteristics, regulations, project objectives, physical sample problems, representativeness, etc. Furthermore, it describes the optimization process of nominated design candidates based on an eight-criterium set. Additionally, the standard provides an overview of the most popular sampling techniques used in environmental and waste management contexts. A comparison between these techniques in terms of their uses, advantages, and limitations is tabulated in Table 2. 

Evidently, this bundle of sampling standards provided by the technical committee D34 are informative and cover a broad range of waste sampling situations. However, they are mostly focused on sampling and sample preparation of environmental waste. As a result, essential material characteristics might be overlooked if they are applied to the plastics recycling context. Furthermore, these guidelines are intended to be followed in tandem since they complement each other. This can substantially increase the complexity of implementation as well as the risk of information loss across the various instructive documents. 

## 3. Derived Sampling Scheme Concept Applied to an In-Plant Case Study

Due to the inconsistencies within the respective standards, selected information from the reviewed standards was used to establish a comprehensive procedure for the sampling and analysis of plastic waste after pretreatment. Typically, in industrial operations of plastics recycling, the materials are directly conveyed to the regranulation process. In such situations, the sampling should be carried out on a time or mass basis on the conveying system. However, in some cases (e.g., pilot plants, small-size plants) materials are delivered discontinuously in the form of pretreated flakes in a big bag (BB) which is then mounted on top of the conveyer belt of the extruder (see Figure 2). Evidently, the flakes are dimensionally heterogeneous as they have different shapes and sizes. This may lead to a substantial material segregation and stratification within a lot or sublot (e.g., BB) with the flakes of smaller size or higher density sinking to the bottom and vice versa [66]. Therefore, a sampling plan for the collection of representative samples from the BBs was proposed in compliance with the operational sequence of ISO 11648–part 2 (see Figure 4). However, the proposed sampling procedure is also relevant for continuous processes by substituting position-based sampling with time-based sampling on the conveyor belt. 

**Figure 7:**
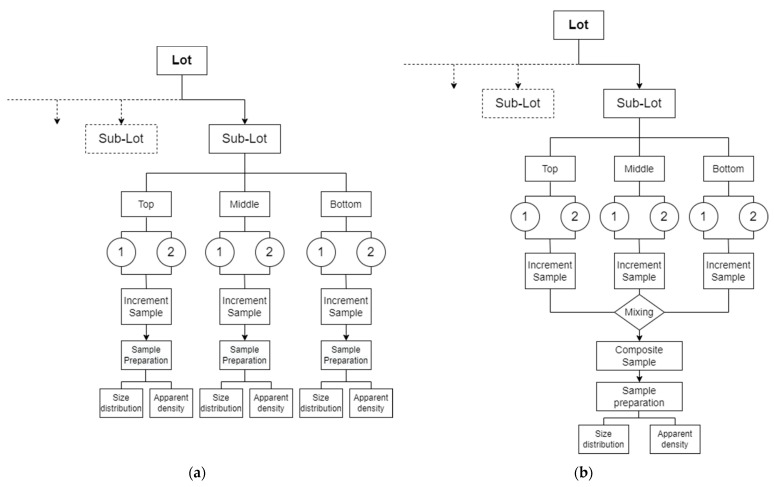
Conception of a sampling scheme for the evaluation of pretreated polyolefin waste based on the operation sequence of the ISO 11648–2:2001, (**a**) variant A which includes the evaluation of the increment samples separately, (**b**) variant B which is based on the evaluation of a composite sample of the whole population.

Stratified random sampling was chosen as the sampling design. The BB was divided into three different strata (bottom, middle, top). Each stratum was divided into two substrata for the collection of the subsamples, which were mixed to generate the increment samples of each stratum. Sampling was performed during the recycling process with the extruder, thus, samples were taken directly from the conveyor belt of the extruder. The subsamples were collected using a metal scoop after different conveyor displacements which represented the different strata positions in the BB. The randomly sampled substratum masses were between 750 g and 1000 g leading to a total mass of 1500 g up to 2000 g of material for each of the three strata. To decide whether to use single or composite samples for the material analysis, two variants of the sampling plan were evaluated based on the respective values of apparent density (AD) and flake size distribution (FSD). Although they are not intrinsic material properties, these two characteristics were selected since they are measures of the material uniformity. Moreover, they are an indication of the handling and processability of the material by delivering information on the ability to feed the material into the extruder [81,82,83]. AD defines the volume occupied by a quantity of particles of a material in a confined space. Typically, PO pellets (i.e., PE and PP) have an AD of approximately 500 up to 580 kg/m^3^ [81,84]. These values fall to as low as 130 and 350 kg/m^3^ in the case of PE films and PP flakes, respectively [84]. Hence, AD strongly varies depending on the form and geometry of the particles of a material and the void entrapped between them [82]. In variant A, the single sample method, increment samples of each stratum were analyzed individually, while in variant B the increment samples were mixed well in a container to produce composite samples. Samples generated by both variants were then analyzed using AD and FSD. Figure 7 shows a schematic illustration of the two sampling plan variants.

### 3.1. Materials and Methods

#### 3.1.1. Pretreated Polyolefin Waste

**Figure 8:**
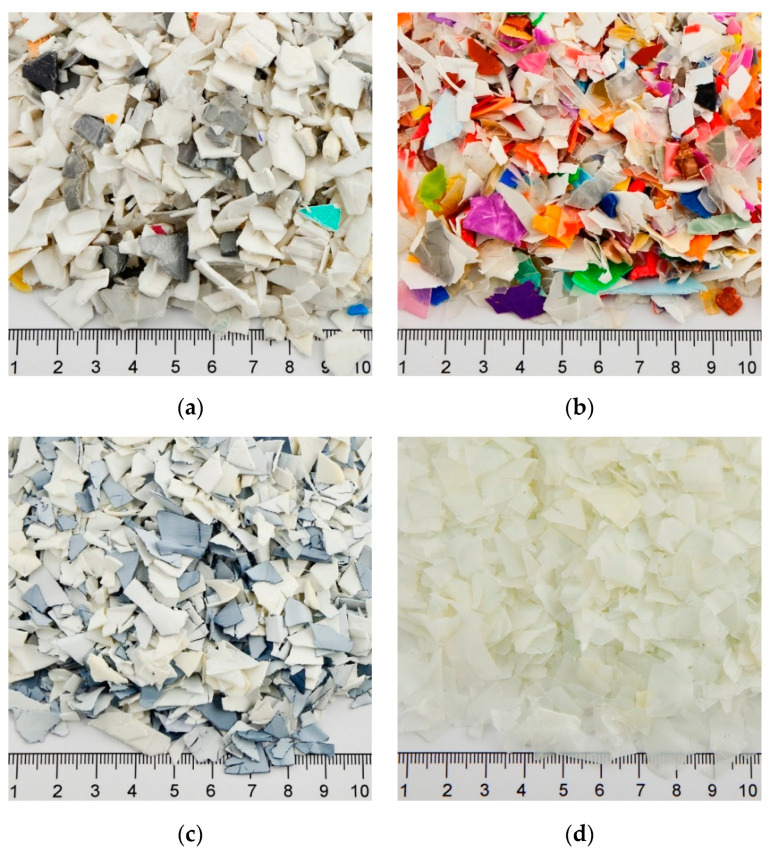
Different pretreated PO waste flakes from postconsumer applications: (**a**) color-sorted rigid PP, (**b**) PP mixture from thin-walled applications, (**c**) PE-HD mixture from multilayer rigid applications, and (**d**) color-sorted PE-HD grade from thin-walled applications.

Four samples of different pretreated postconsumer polyolefin (PO) waste from packaging applications were chosen for this study. All material feedstocks were supplied in a BB of approximately 700 kg each. Two of them were polypropylene (PP) dominated, while the other two were high-density polyethylene (PE-HD)-dominated mixtures. Top view pictures of samples of the materials are shown in Figure 8. 

**PP:** the first PP grade (PP_1) presumably came from rigid applications as the flakes of this material had an average thickness of 2.1 ± 0.8 mm. The average thickness of the second PP grade (PP_2) was around 0.7 ± 0.2 mm, thus it can be assumed that it originated from thin-walled packaging applications. The former grade was color-sorted, whereas the latter was a mixture of colors.

**PE-HD:** similarly, the PE-HD grades were selected to cover different segments of the packaging market. Hence, the first grade (PE-HD_1) had a thickness of 1.3 ± 0.6 mm and originated from multilayer applications with a combination of grey and white colors, while the other grade (PE-HD_2) had an average thickness of 0.6 ± 0.2 mm and a natural color.

#### 3.1.2. Flake Size Distribution

The flake size distribution (FSD) was determined according to the test method B in the standard guide ASTM D1921 [83], which is directed at materials with irregular particle size distribution. The analysis was carried out by an automatic vibratory sieve shaker (model AS 200 control B) manufactured by RETSCH GmbH (Haan, Germany) with defined particle size ranges. Due to the asymmetric geometry of plastic flakes, a particle is considered from a size span once two of its dimensions fall into the respective range. The screen sizes were chosen to cover the ranges of interest as 2.0, 4.0, 8.0, and 11.2 mm. The sieves were stacked with the coarsest sieve on top to the finest on the bottom pan. After mixing properly, a sample of approximately 1000 g of every increment and composite sample was collected for the analysis. Each sample was divided into portions of 100 ± 5 g to avoid clogging the screens. Finally, the retained material fractions on each sieve screen were weighed to the nearest 0.01 g by a digital laboratory scale. These weights were then used to calculate the percentage of each size span.

#### 3.1.3. Apparent Density

The apparent density (AD) of the four different feedstocks was determined based on test method C in the standard guide [82]. This method is specifically designed for molding materials that are supplied in irregular forms, such as flakes; chips; fiber cuts, etc. The measurement tool consists of a cylinder with a volume of 1000 cm^3^ and a hollow cylinder closed at one end (plunger) with a slightly smaller outer diameter than the inner diameter of the first cylinder. The tool was self-constructed by 3D printing of PLA material with fused deposition modelling technology. To measure AD, the measuring cylinder is placed on a flat surface and then 60.0 ± 0.2 g of the material is poured into it. Afterwards, the plunger is loosely placed onto the material to measure the height of the volume that the material occupies in the cylinder. The measurement is performed without and with a weight shot of 2300 ± 20 g (including the weight of the plunger). The additional weight can be inserted into the plunger. After measuring the heights (*h*_1_) and (*h*_2_), without and with the weight load, respectively, AD is calculated as:

(1) $$V=h\times A\;\mathrm{AD}=\frac{W}{V}\;\;\;\;\;\;\;\;\;or\;\;\;\;\;\;\mathrm{AD}=\frac{W}{h\times A}$$

where: *V* is the occupied volume in the cylinder, *h* is the height of the material in the cylinder, *A* is the inner cross-section area of the cylinder, *W* is the weight of the poured material.

### 3.2. Results and Discussion

#### 3.2.1. Effect of the Sampling Position on the Fraction Size Distribution

**Figure 9:**
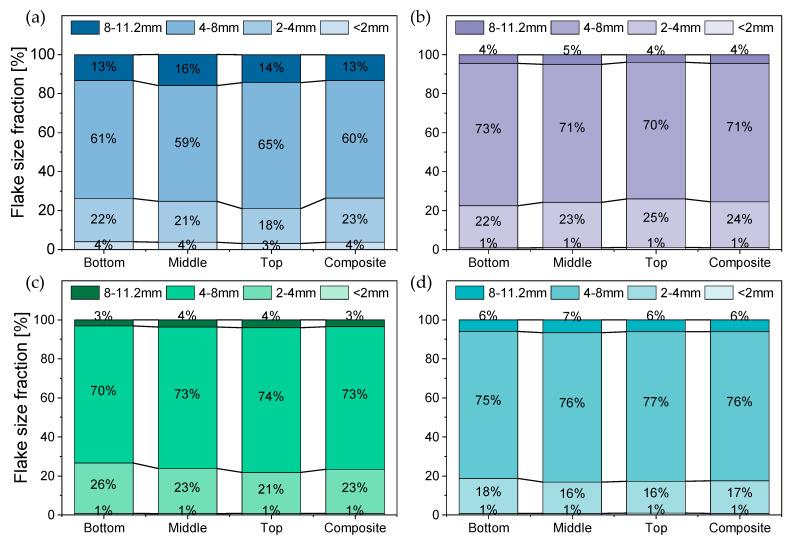
Flake size distribution (FSD) of the various PO feedstocks based on the evaluation by a vibratory sieve shaker tower. (**a**) PP_1, (**b**) PP_2, (**c**) PE-HD_1, (**d**) PE-HD_2.

Figure 9 illustrates the results of the FSD of the different pretreated PO feedstocks, which were obtained by the analysis of the retained size fractions on the sieve screens of the vibratory shaker. Coarse flakes with size of 11.2 mm or above were completely absent in the samples of the four materials. The size span (4.0–8.0 mm) was predominant in all analyzed samples (increment and composite) followed by (2.0–4.0 mm), then (8.0–11.2 mm), and lastly the fine fraction (flake size < 2 mm) as the smallest size fraction. Around 60% of the flakes of material PP-A had a size between 4 and 8 mm (Figure 9a). This proportion increases in the other three materials to account for over 70% in PP-B and PE-HD_1 and over 75% in PE-HD_2. In contrast, the fine fraction accounted for approximately 4% in PP-A in both increment and composite samples, whereas only 1% or below of the other three materials happened to be of the fine fraction (<2 mm). Therefore, the impact of this fraction was neglected in other measurements of these materials as the collected amount was not sufficient (<60 g) to perform the apparent density measurements.

Overall, all four feedstocks exhibited the same trend within the different strata as only slight variations in the FSD of the different positions could be observed. Furthermore, the FSD of the composite samples also showed a comparable behavior to that of the increment samples. Hence, it can be inferred that the analysis of a composite sample provides sufficient representative information about the FSD of a pretreated PO feedstock in a BB. 

#### 3.2.2. Effect of Sampling Position on the Apparent Density

**Figure 10:**
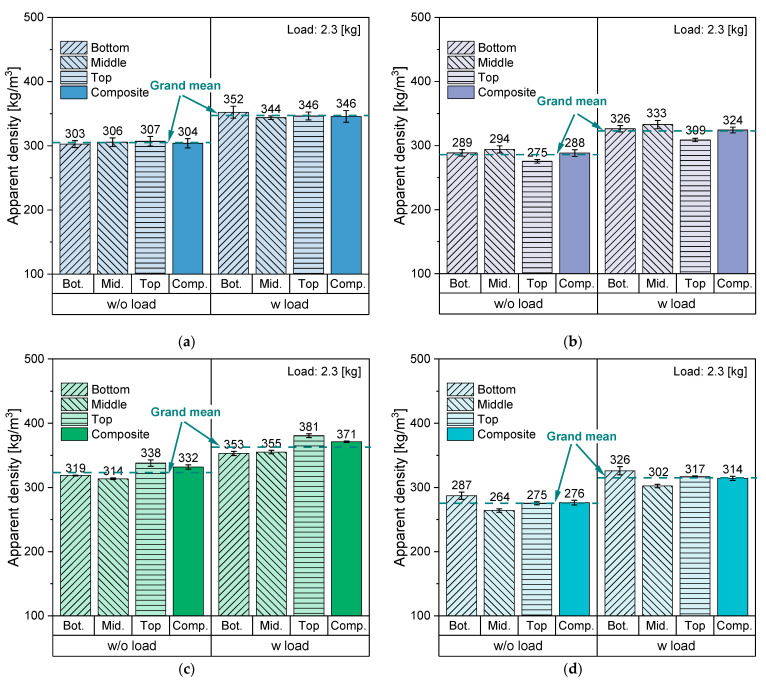
AD values of the individual increment and composite samples of four PO feedstocks without and with weight load based on the test method from ASTM D1895 [82]. (**a**) PP_1, (**b**) PP_2, (**c**) PE-HD_1, (**d**) PE-HD_2.

Figure 10 shows the average AD values together with standard deviations of increment and composite samples of the four PO materials without and with weight load. To estimate the precision of the measurement and to ensure a good representation of the samples, the measurement was performed on three randomly extracted increment and composite samples and then the average and the standard deviation were calculated. In general, the rigid flakes had a higher AD than that of the thin-walled ones regardless of the PO type, although the AD of the PE-HD_1 was slightly higher than that of the other investigated grades. This can be explained by the slightly higher density of the multilayer PE-HD in comparison to PO grades. For the variability and the significance check, several statistical parameters (e.g., mean, standard deviation, variance) were evaluated. The measurements of the individual samples of all four materials yielded a small standard deviation within each position, thus, the measurement error can be presumed insignificant. When comparing the three sampling positions, PP_1 showed the smallest feedstock variability as the average values of its individual samples were around the grand mean (GM) with overlapping standard deviations (Figure 10a). This can be attributed to the geometry of its flakes and to the relatively higher fine fraction in its composition, which may have led to filling the empty volumetric spaces in the measurement cylinder, hence more unified AD values. However, a more noticeable variation between the sampling positions could be observed in the other three materials.

Analysis of variance (ANOVA) was carried out to statistically evaluate the significance of this observation. The probability value (*p*-value) is usually used to reject or accept the null-hypothesis (H_0_), which states that the mean values of at least two groups are more likely to be equal. If the *p* value is smaller than the significance level (α), which is typically 0.05, the result is deemed to have a statistical significance, hence the null-hypothesis is rejected. In contrast, if the *p*-value is greater than α, H_0_ has a higher probability to have occurred.

Based on the ANOVA results in Table 3, the observation can be confirmed. On the one hand, for PP_1 the *p*-value of AD was higher than α in both cases (without and with load), thus H0 cannot be rejected. This confirmed the lower feedstock variability between the three sampling positions in terms of AD. On the other hand, the *p*-value of the other three materials—PP_2, PE-HD_1, PE-HD_2—was approaching zero. Hence, the results had a statistical significance, which gave sufficient evidence to reject H_0_ and proved the high variability between the three sampling positions. Nevertheless, the individual values of the increment samples of each material can be grouped together to calculate GM and the overall standard deviation (also known as pooled or combined standard deviation). This provides one single value that presumably represents the whole bag and can be used for the comparison between the two proposed sampling variants.

**Figure 11:**
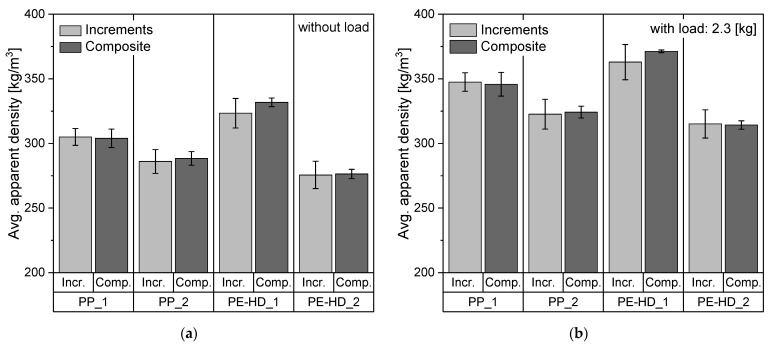
A comparison between the average values of the grouped increment samples and the respective composite samples of the PO feedstocks based on the analysis of variance (ANOVA). (**a**) average AD values of loose samples without a weight load, (**b**) average AD values of compacted samples with a weight load of 2.3 kg.

Figure 11 depicts the GM of the increment samples and the average values of the composite samples. Evidently, both sampling methods eventually led to comparable AD values in all materials, although, in most cases, the composite sample had a notably smaller standard deviation than that of the grouped increments. This could be attributed to the better homogenization of the sample through proper mixing as well as the reduced number of measurements, which prevented higher possibility of introducing a systematic measuring error. Furthermore, the ANOVA results given in Table 4 show the *p*-values of the four materials under both measuring conditions. All *p*-values were higher than α, hence, there is not enough evidence to reject H_0_ at a level of significance of 5%. Therefore, the likelihood of both sampling variants having equal mean values is high. This leads to the conclusion that, unlike the collection of single random samples, composite sampling generates representative samples of feedstocks with high variability. Hence, composite samples convey true material characteristics.

Furthermore, the insertion of the load shot into the plunger increased the AD values of the four materials due to the compaction of the flakes inside the cylinder. To quantify the increase in AD, the compaction factor (*f_c_*)—the ratio between the two apparent densities—of the samples was calculated as follows:

(2) $$f_{C}=\frac{{\mathrm{AD}}_{2}}{{\mathrm{AD}}_{1}}$$

where: AD_1_ is the apparent density without a load shot and AD_2_ is the apparent density with a load.

As shown in Table 5, the values of *f*_c_ slightly varied between the three strata in all feedstocks. However, the average values of the three positions were identical to that of the composite sample in PP_1 and both PE grades. The individual values of PP_2 were slightly higher than *f*_c_ of its composite sample, thus, did not yield the same trend as the other materials. This might be due to either the geometry of the flakes of this material or a systematic error in the measurement itself. 

#### 3.2.3. Effect of the Flake Size on the Apparent Density

After sieving the materials as described in Section 3.1.2, AD measurement was performed on the retained size fractions of the composite samples to understand the relationship between the flake size and AD. The measurement was performed in accordance with the test method described in Section 3.1.3. To ensure an adequate level of accuracy, three measurements were performed on each size fraction and then the individual average and standard deviation of each size fraction were calculated. Additionally, to have a better understanding of the formation of the composite samples, the collective AD of all size fractions was calculated based on their individual values and proportions in the composite sample as follows:

(3) $${\mathrm{AD}}_{cal}=\overset{n}{\underset{i}{\sum }}R_{i}.{\mathrm{AD}}_{i}$$

where: *R_i_* is the ratio of a size fraction in the composite sample and AD*_i_* is the corresponding apparent density of that size fraction.

**Figure 12:**
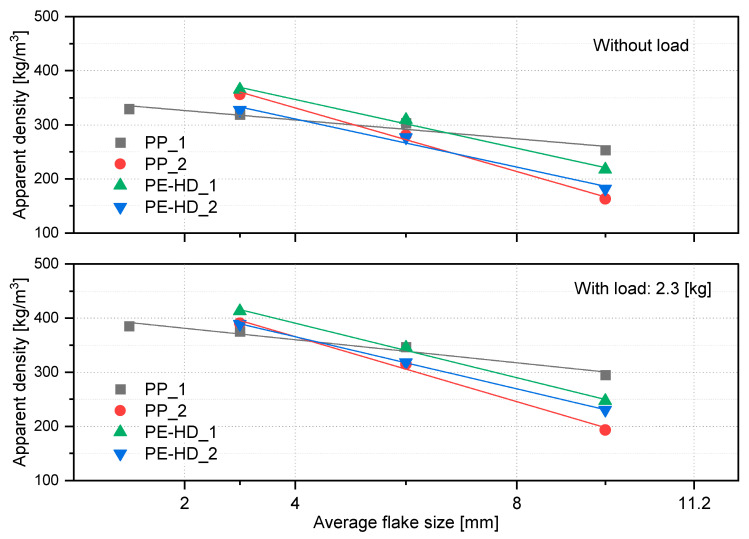
A correlation between the mean values of the flake size fractions and the corresponding AD values of the investigated size spans.

**Figure 13:**
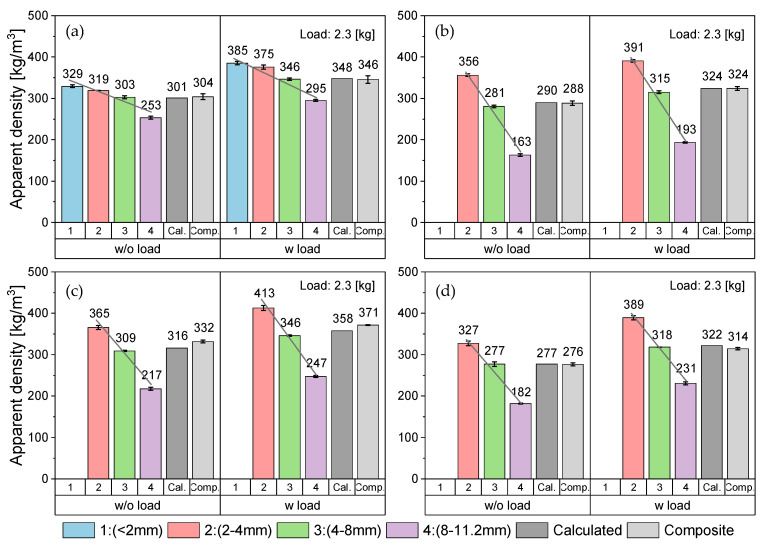
AD without and with weight load based on the test method from ASTM D1895 [82] of the various size fractions in comparison to the values of their corresponding calculated AD and composite samples (**a**) PP_1, (**b**) PP_2, (**c**) PE-HD_1, (**d**) PE-HD_2.

By plotting the mean values of the various size spans against the respective AD values, a linear correlation between AD and the flake size can be derived (Figure 12). It was evident that in all four materials and under both measurement conditions, the AD value sank with increasing the flake size, and vice versa. On the other hand, Figure 13 shows a more detailed comparison of the individual AD values of the different size fractions along with their calculated AD, as well as the values of the corresponding composite samples. The fine fraction (<2 mm) was excluded from this part of the study for PP_2 and both PE-HD grades. This is because only an insufficient amount of this fraction was found in the samples (<1%), which hindered the measurement, and thus the impact of this fraction was neglected. However, the overall material behavior was not considerably affected by the lack of information on this fraction. The difference between the various size fractions was most prominent in PP_2 (Figure 13b). For this material, the AD values of the biggest size span (8.0–11.2 mm) were over 50% and 40% lower than those of the finest considered fraction (2.0–4.0 mm) and the composite sample, respectively. In contrast, PP_1 had the least significant difference between the different size spans as the AD values of the biggest span were only 23% and around 15% lower than those of the fine fraction and the composite sample, respectively (Figure 13a), whereas the AD values of both PE-HD grades declined by up to 44% with the biggest flake size fraction (Figure 13c,d).

Furthermore, the calculation of AD, according to Equation (3), yielded values in the same range as the measurement of the composite samples with only a small margin of error. The error between the two sources was below 1% for all materials under both measurement conditions except for PE-HD_1 without load and PE-HD_2 with load, where it accounted for approximately 1.5% and 2.5%, respectively. This slightly higher error can be attributed to a systematic error in the measurement itself or to the lack of information about the AD of the fine fraction, which may have affected the accuracy of the results. 

Nevertheless, based on the overall results of the investigated materials, it can be concluded that composite sampling provides representative samples of a feedstock with variable particle sizes and proportions. Hence, the analysis of such samples can sufficiently convey the true material characteristics with a good level of accuracy.

## 4. Conclusions and Outlook

The first part of the paper presented a review of the available sampling methods in the field of plastics recycling as well as other methods that can be applicable in such a context with a short summary of various relevant standards. It was found that, on the one hand, the literature only offers composition studies of household waste to provide statistical information on the topic. However, there have not yet been any studies dealing with the topic from an engineering perspective. From the three most prominent standardization organizations, only DIN established a two-part sampling practice directed at the plastics recycling industry. Overall, neither in literature nor in the standards was a clear operational sequence for sampling and further basic characterization presented for proper use in the plastics recycling industry.

In the second part of the paper, a concept for a sampling plan was introduced in accordance with the recommended operational sequence of ISO–11648 and with information from other standards. Two variants of the sampling plan were evaluated based on flake size distribution and apparent density. Stratified random sampling was selected as the sampling technique. In the first variant, increment samples were evaluated individually, while in the second, a well-mixed composite sample was evaluated. The results of the study showed an agreement that stratified random sampling in combination with composite sampling provide a good sampling technique for PO waste. Based on the analysis of variance results, the analysis of composite samples sufficiently provides representative information of the true material characteristics. Moreover, it also offers higher efficiency due to the reduced number of investigated samples and testing time. In conclusion, the authors recommend time-based collection of increment samples to form the necessary composite samples, which can be used for QC purposes, at the various processing steps in the case of continuous processing in plastics recycling plants. 

The aim of the present paper was to shed a light on the lack of standardized methods for sufficient sampling in the field of plastics recycling and to address the importance of sampling for the generation of representative data of such heterogeneous material streams. Furthermore, this study was an attempt to build a fundamental sampling procedure based on simple characterization methods, which can serve in more advanced studies in the future. Hereafter, it is intended to conduct more advanced studies towards the development of a comprehensive quality assurance scheme that ensures proper data acquisition throughout the whole value chain of plastics recycling.

## Figures and Tables

**Table 1:** Relevant standardized methods for the sampling of plastic waste (compiled by authors).

Institution	Method	Reference
The International Organization for Standardization (ISO)	Statistical aspects of sampling from bulk materials: Part 1 General principles	ISO 11648-1
	Statistical aspects of sampling from bulk materials: Part 2 Sampling of particulate materials	ISO 11648-2
Deutsches Institut für Normung (DIN)	Sampling procedures for testing plastics waste and recyclate	DIN CEN/TS 16010
	Recycled plastics—Sample preparation	DIN CEN/TS 16011
American Society for Testing and Materials (ASTM)	Standard Guide for General Planning of Waste Sampling	ASTM D4687
	Sampling strategies for heterogeneous wastes	ASTM D5956
	Standard Guide for Sampling Waste Piles	ASTM D6009
	Representative sampling for management of waste and contaminated media	ASTM D6044
	Composite sampling and field subsampling for environmental waste management activities	ASTM D6051
	Generation of environmental data related to waste management activities: selection and optimization of sampling design	ASTM D6311
	Sampling waste streams on conveyors	ASTM D7204

**Table 2:** A summary of advantages and limitations of the most common sampling designs from ASTM D6311 [80].

Sampling Design	Uses	Advantages	Limitations
Authoritative Judgment	estimate of population mean when population is homogeneous when high margin of error is acceptable when sampling designer has knowledge	cost effective	if population is heterogeneous, the mean is not easily estimated has high margin of error poor estimate of variance
Biased	ID localized contamination Determine non-compliance	cost effective	cannot generalize to the entire population
Simple Random	when population is not stratified	simple estimates variance	if stratified populations, may not estimate mean accurately need more samples may not be easy logistically
Stratified Random	when population can be divided into relatively homogeneous strata	when the resulting strata are homogeneous representative samples estimates variance	may be difficult logistically strata must correctly reflect any contaminant stratification
Systematic Line Space Random	locate hot spots map trends	samples easily identified and collected can define contamination patterns more accurate estimate of mean concentration	unrecognized trends or cycles may cause poor accuracy or precision, or both
Search	locate hot spots	cost effective minimum samples needed easy to implement	hot spot may be undetected
Unequal Probability	heterogeneous population contaminant expected in specific fraction	more precise estimate of the chemical contamination in a heterogeneous material less costly	unrecognized trends or cycles may cause poor accuracy or precision, or both

**Table 3:** Analysis of variance (ANOVA–single factor) within the grouped increment samples of the four PO feedstocks based on AD with a level of significance (α = 0.05).

Material	Apparent Density	SS ^1^	MS ^2^	F ^3^	*p*-Value
PP_1	*w*/*o* load	5.1 × 10^−5^	2.5 × 10^−5^	0.5682	0.58
	*w* load	1.9 × 10^−4^	9.5 × 10^−5^	2.1484	0.16
PP_2	*w*/*o* load	5.5 × 10^−4^	2.7 × 10^−4^	12.283	0.01
	*w* load	9.2 × 10^−4^	4.6 × 10^−4^	18.529	0.00
PE-HD_1	*w*/*o* load	9.9 × 10^−4^	4.9 × 10^−4^	54.663	0.00
	*w* load	1.4 × 10^−3^	7.2 × 10^−4^	76.707	0.00
PE-HD_2	*w*/*o* load	8.0 × 10^−4^	4.0 × 10^−4^	27.010	0.00
	*w* load	8.6 × 10^−4^	4.3 × 10^−4^	26.280	0.00

^1^ SS: sum of squares, ^2^ MS: mean square, ^3^ F: F ratio.

**Table 4:** Analysis of variance (ANOVA–single factor) between the grouped increment samples and their counterpart composite samples of the four PO feedstocks based on AD with a level of significance (a = 0.05).

Material	Apparent Density	SS ^1^	MS ^2^	F ^3^	*p*-Value
PP_1	*w*/*o* load	2.9 × 10^−6^	2.9 × 10^−6^	0.0679	0.80
	*w* load	7.4 × 10^−6^	7.4 × 10^−6^	0.1333	0.72
PP_2	*w*/*o* load	1.2 × 10^−5^	1.2 × 10^−5^	0.1692	0.69
	*w* load	6.0 × 10^−6^	6.0 × 10^−6^	0.0542	0.82
PE-HD_1	*w*/*o* load	1.6 × 10^−4^	1.6 × 10^−4^	1.4913	0.25
	*w* load	1.6 × 10^−4^	1.6 × 10^−4^	1.0432	0.33
PE-HD_2	*w*/*o* load	1.3 × 10^−6^	1.3 × 10^−6^	0.0146	0.91
	*w* load	1.8 × 10^−6^	1.8 × 10^−6^	0.0184	0.89

^1^ SS: sum of squares, ^2^ MS: mean square, ^3^ F: F ratio.

**Table 5:** List of the compaction factors of the four feedstocks.

Material	Bottom	Middle	Top	Average	Composite
PP_1	1.16	1.13	1.13	1.14	1.14
PP_2	1.13	1.13	1.12	1.13	1.12
PE-HD_1	1.11	1.13	1.13	1.12	1.12
PE-HD_2	1.14	1.14	1.15	1.14	1.14

## Data Availability

The data presented in this study are available on request from the corresponding author.

## List of Abbreviations

ASTM	American Society for Testing and Materials
ANOVA	Analysis of variance
AD	Apparent density
BB	Big bag
CO_2_	Carbon dioxide
CE	Circular economy
f_c_	Compaction factor
DIN	Deutsches Institut für Normung
F	F ratio
FSD	Flake size distribution
GM	Grand mean
PE-HD	High-density polyethylene
ICS	Informal collection system
ISO	International Organization for Standardization
MS	Mean square
MFR	Melt flow rate
Mt	Million metric tons
NAFTA	North American Free Trade Agreement (Mexico, Canada, and the United States)
H_0_	Null-hypothesis
PET	Polyethylene terephthalate
PE	Polyethylene
PO	Polyolefin
PP	Polypropylene
PVC	Polyvinyl chloride
PCPW	Postconsumer plastic waste
PIPW	Postindustrial plastic waste
*p*-value	Probability value
QC	Quality control
RP	Rigid packaging
α	Significance level
SS	Sum of squares
TC	Technical committee
TOS	Theory of sampling
